# The identification of gene signatures in patients with extranodal NK/T-cell lymphoma from a pair of twins

**DOI:** 10.1186/s12885-021-09023-9

**Published:** 2021-12-06

**Authors:** Yang Wang, Huaicheng Tan, Ting Yu, Xuelei Ma, Xiaoxuan Chen, Fangqi Jing, Liqun Zou, Huashan Shi

**Affiliations:** 1 Laboratory of Aging Research and Cancer Drug Target, State Key Laboratory of Biotherapy, National Clinical Research Center for Geriatrics, West China Hospital, Sichuan University, No. 17, Block 3, Southern Renmin Road, Chengdu, Sichuan 610041 PR China; 2 Department of Pathology and Laboratory of Pathology, State Key Laboratory of Biotherapy, West China Hospital, West China School of Medicine, Sichuan University, Chengdu, China; 3 Department of Biotherapy, Cancer Center, West China Hospital, Sichuan University, Chengdu, China; 4 Department of Radiotherapy, Cancer Center and State Key Laboratory of Biotherapy, West China Hospital, Sichuan University, Chengdu, China; 5 State Key Laboratory of Oral Diseases, National Clinical Research Center for Oral Diseases, West China Hospital of Stomatology, Sichuan University, Chengdu, 610041 China; 6 Department of Head and Neck Cancer, Cancer Center, West China Hospital, Sichuan University, Chengdu, China

**Keywords:** Extranodal NK/T-cell lymphoma, Sequencing, Support vector machine-recursive feature elimination, Machine learning algorithms, Single sample gene set enrichment analysis, Immune infiltration

## Abstract

**Background:**

There is no unified treatment standard for patients with extranodal NK/T-cell lymphoma (ENKTL). Cancer neoantigens are the result of somatic mutations and cancer-specific. Increased number of somatic mutations are associated with anti-cancer effects. Screening out ENKTL-specific neoantigens on the surface of cancer cells relies on the understanding of ENKTL mutation patterns. Hence, it is imperative to identify ENKTL-specific genes for ENKTL diagnosis, the discovery of tumor-specific neoantigens and the development of novel therapeutic strategies. We investigated the gene signatures of ENKTL patients.

**Methods:**

We collected the peripheral blood of a pair of twins for sequencing to identify unique variant genes. One of the twins is diagnosed with ENKTL. Seventy samples were analyzed by Robust Multi-array Analysis (RMA). Two methods (elastic net and Support Vector Machine-Recursive Feature Elimination) were used to select unique genes. Next, we performed functional enrichment analysis and pathway enrichment analysis. Then, we conducted single-sample gene set enrichment analysis of immune infiltration and validated the expression of the screened markers with limma packages.

**Results:**

We screened out 126 unique variant genes. Among them, 11 unique genes were selected by the combination of elastic net and Support Vector Machine-Recursive Feature Elimination. Subsequently, GO and KEGG analysis indicated the biological function of identified unique genes. GSEA indicated five immunity-related pathways with high signature scores. In patients with ENKTL and the group with high signature scores, a proportion of functional immune cells are all of great infiltration. We finally found that CDC27, ZNF141, FCGR2C and NES were four significantly differential genes in ENKTL patients. ZNF141, FCGR2C and NES were upregulated in patients with ENKTL, while CDC27 was significantly downregulated.

**Conclusion:**

We identified four ENKTL markers (ZNF141, FCGR2C, NES and CDC27) in patients with extranodal NK/T-cell lymphoma.

## Introduction

Extranodal NK/T-cell lymphoma (ENKTL) is a subtype of non-Hodgkin lymphoma characterized by progressive lesions in nasal cavities, the middle of the face, upper aerodigestive tracts and other non-nasal sites. The disease frequently occurs in Asian and Latin Americans [1]. The infection of Epstein-Barrvirus (EBV) may be closely related to its pathogenesis [2]. At an early stage of ENKTL, the combination of chemotherapy and radiotherapy prolongs patients’ survival and improves the quality of life [3, 4]. However, for advanced refractory ENKTL patients, the efficacy of current treatment is not satisfactory [5]. Immunotherapy provides a new direction for these patients [6, 7]. Immunotherapy for programmed cell death protein 1 (PD-1) and programmed cell death protein ligand 1 (PD-L1) has enormously improved the therapeutic effect of ENKTL [8, 9]. Searching for tumor-specific genes is beneficial for ENKTL diagnosis, the discovery of tumor-specific neoantigens and the development of novel therapeutic strategies. These tumor-specific genes can be used as predictors of the prognosis. Nevertheless, the genetic landscape and the mutation signature of ENKTL remain to be elucidated.

By understanding the existence of the tumor-assocoated unique genes, we could enrich therapeutic methods to improve the prognosis. A good illustration is epidermal growth factor receptor (EGFR)/anaplastic lymphoma kinase (ALK) in lung cancer [10], CD19 in diffuse large B cell lymphoma [11] and HER2 in breast cancer [12]. Recently, gene detection has been a predictor for prognosis and treatment sensitivity of cancer patients. As for ENKTL, gene expression profiling (GEP) identified unique signatures which are mainly from neoplastic NK cells. Cytotoxic-molecule (granzyme H) levels and the activity of ENKTL signaling pathways (NF-κB and JAK/STAT3) are both elevated [8, 13]. Some gamma delta-peripheral T cell lymphomas (γδ-PTCLs) have STAT3 mutations [14]. Except for the above features, a genetic investigation found 6q21 deletion and PRDM1 as a candidate gene in NK cell-related malignancies. PRDM1 locates at the minimal common region (MCR). The methylation of PRDM1 inhibits PRDM1 expression [15]. When treated with decitabine, NK cells would experience toxicity by enhancing PRDM1 levels [16]. Therefore, The methylation of PRDM1 maybe exists in ENKTL. HACE1 is another gene located within the 6q21 region. The loss of HACE1 function is realized by the deletion and hypermethylation of cytosine phosphate guanine island. The abnormal HACE1 within 6q21 is a cause of NK cell lymphomagenesis [17].

Machine learning algorithms are now involved in numerous aspects of medical studies, which integrate AI tools into clinical practice. As for medicine, ML is a scientific tool to analyze large-scale data appropriately [18, 19]. It fosters us to understand cancer comprehensively from molecular perspectives, especially its cancer-diagnosis application [20–22]. Therefore, ML is valuable to find out valuable biomarkers in multiple data. In ML, support-vector machines (SVMs) are significant learning models with algorithms for classification and regression analysis. They can select biomarkers that are the most effective classification [23, 24].

Our study aims at identifying gene signatures in patients with extranodal NK/T-cell lymphoma. Initially, we detected genes from a pair of twins with ENKTL and analyzed unique differential genes. Based on these genes, we analyzed ENKTL patients’ information in several databases to predict specific antigen mutations and new targets. We hope to understand the genetic background and to seek for targets to predict prognosis. Therefore, the understanding of ENKTL’s genetic background would benefit us enormously.

## Materials and methods

### Data collection and sample cluster

**Fig. 1:**
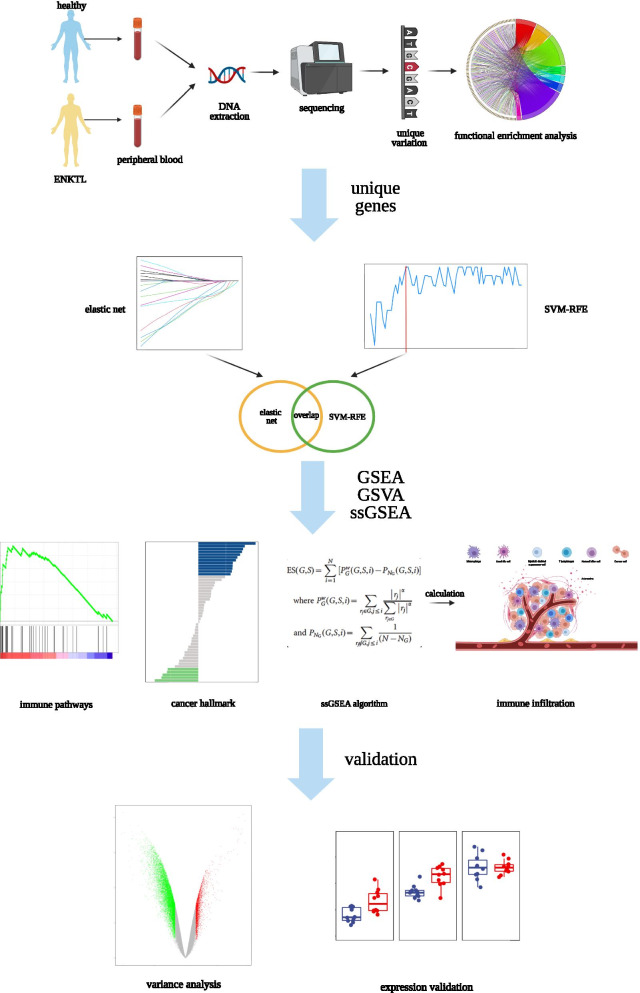
Flow diagram of the procedure. First, from the peripheral blood of a pair of twins, we applied a whole-genome shotgun (WGS, Beijing Boao Biological Co., Ltd) for sequencing to identify unique variant genes. One individual is diagnosed with ENKTL, while the other is healthy. Next, we downloaded a training dataset (GSE 80632) and testing database (GSE 19067) from Gene Expression Omnibus (GEO) (https://www.ncbi.nlm.nih.gov/gds/). Subsequently, we conducted the Robust Multi-array Analysis (RMA) and z score normalization to preprocess the data. To understand the biological function of unique mutated genes, GO (Gene Ontology) enrichment analysis and KEGG (Kyoto Encyclopedia of Genes and Genomes) enrichment analysis were performed in DAVID (https://david.ncifcrf.gov/). R package goplot was used for visualization. Then, to select unique genes, we used an elastic net to fit a generalized linear model by the R package glmnet and analyzed the training dataset by using the elastic net. Simultaneously, we used another algorithm called Support Vector Machine-Recursive Feature Elimination (SVM-RFE) to identify unique genes. Nest, to explore pathway gene sets of selected markers, we conducted GSEA and GSVA of the training set data. We performed GSEA with GSEA V4.1.0 software. Correspondingly, GSVA relied on R package “GSVA”. We conducted single-sample gene set enrichment analysis (ssGSEA) to achieve enrichment scores of immune-filtrating cells by calculating enrichment scores. Also, we performed Spearman correlation tests to assess correlation and used R package pheatmap for visualization. Fianally, to validate the reliability and accuracy of unique genes, the validation set was used to verify the expression of the screened markers. For differential genes, we used Boxvolin plots to demonstrate their expression levels. Created with BioRender.com

The procedure of our study is illustrated in Fig. 1. First, from the peripheral blood of a pair of twins, we applied a whole-genome shotgun (WGS, Beijing Boao Biological Co., Ltd) for sequencing to identify unique variant genes. One individual is diagnosed with ENKTL, while the other is healthy. WGS relies on the Illumina NovaSeq 6000 sequencing system. The sequence libraries for the system are composed of conventional small DNA fragments from genomic DNA samples. The end-repair of DNA fragments was added an ‘A’ base at the 3′-end of each strand, followed by the ligation-mediated PCR, single strand separation and cyclization. DNA Nanoballs (DNBs) was produced by the rolling circle amplification, being loaded into nanoarrays and processed for 100 bp pair-end sequencing. The mothod has a 30× sequencing depth and data size of 90G [25]. Next, we downloaded a training dataset (GSE 80632) from Gene Expression Omnibus (GEO) (https://www.ncbi.nlm.nih.gov/gds/) based on the platform GPL6883 from Illumina HumanRef-8 v3.0 expression beadchip. The database contains 25 ENKTL tissues and 15 normal tissues. Similarly, our testing database (GSE 19067) is from GEO, containing 21 ENKTL samples and 11 NK-cell lines. Subsequently, we conducted the Robust Multi-array Analysis (RMA) and *z* score processing to preprocess the normalized data [26]. Although these two databases contain different sets of genes, they both contain unique mutated genes which was sequenced by WGS.

### Functional enrichment analysis

To understand the biological function of unique mutated genes, GO (Gene Ontology) enrichment analysis and KEGG (Kyoto Encyclopedia of Genes and Genomes) enrichment analysis were performed in DAVID (https://david.ncifcrf.gov/). R package goplot was used for visualization.

### Identification of unique genes for ENKTL

To select unique genes, we used an elastic net to fit a generalized linear model by the R package glmnet and analyzed the training dataset by using the elastic net [27, 28]. We performed leave-one-study-out cross validation for classification validation and selected a penalty of 0.6 to fit a generalized linear model. Simultaneously, we used another algorithm called Support Vector Machine-Recursive Feature Elimination (SVM-RFE) to identify unique genes by applying *e1071* package [29]. Next, we combined unique genes from the elastic net and the SVM-RFE algorithms and selected a total of 11 signature genes for further validation. Finally, we calculated patients’ signature scores to evaluate the biological difference of patients who had different unique genes (signature scores = ∑i Coefficient (mRNAi) × Expression (mRNAi)).

### Gene set enrichment analysis (GSEA) and gene set variation analysis (GSVA)

To explore pathway gene sets of selected markers, we conducted GSEA and GSVA of the training set data. Based on the median of signature scores, samples were divided into the high group and the low group. We performed GSEA with GSEA V4.1.0 software in which *c2.cp.kegg.v7.2.symbols.gmt* serves as our defined background set of genes to be tested for significant and concordant differences between two biological states. Correspondingly, GSVA relied on R package “GSVA” in which *h.all.v7.4. symbols.gmt* serves as our defined background set of genes to be tested for significant and concordant differences between two biological states. R package (Limma) is used to calculate differences.

### Immune infiltration analysis

We conducted single-sample gene set enrichment analysis (ssGSEA) [30] to achieve enrichment scores of immune-filtrating cells by calculating enrichment scores that stand for absolute enrichment levels of a gene set in a sample. Then, we performed Spearman correlation tests to assess correlation and used R package pheatmap for visualization.

### Validation of signature genes

To validate the reliability and accuracy of unique genes, the validation set was used to verify the expression of the screened markers. Limma packages were used to calculate differential genes between the normal group and ENKTL group. We defined *lg*|*fc*| *> 1* and *adj.pvalue < 0.05* as significant difference. For differential genes, we used Boxvolin plots to demonstrate their expression levels. Wilcox test is responsible for detecting statistical differences.

## Results

### Sequencing of twins’ unique variant genes

**Fig. 2:**
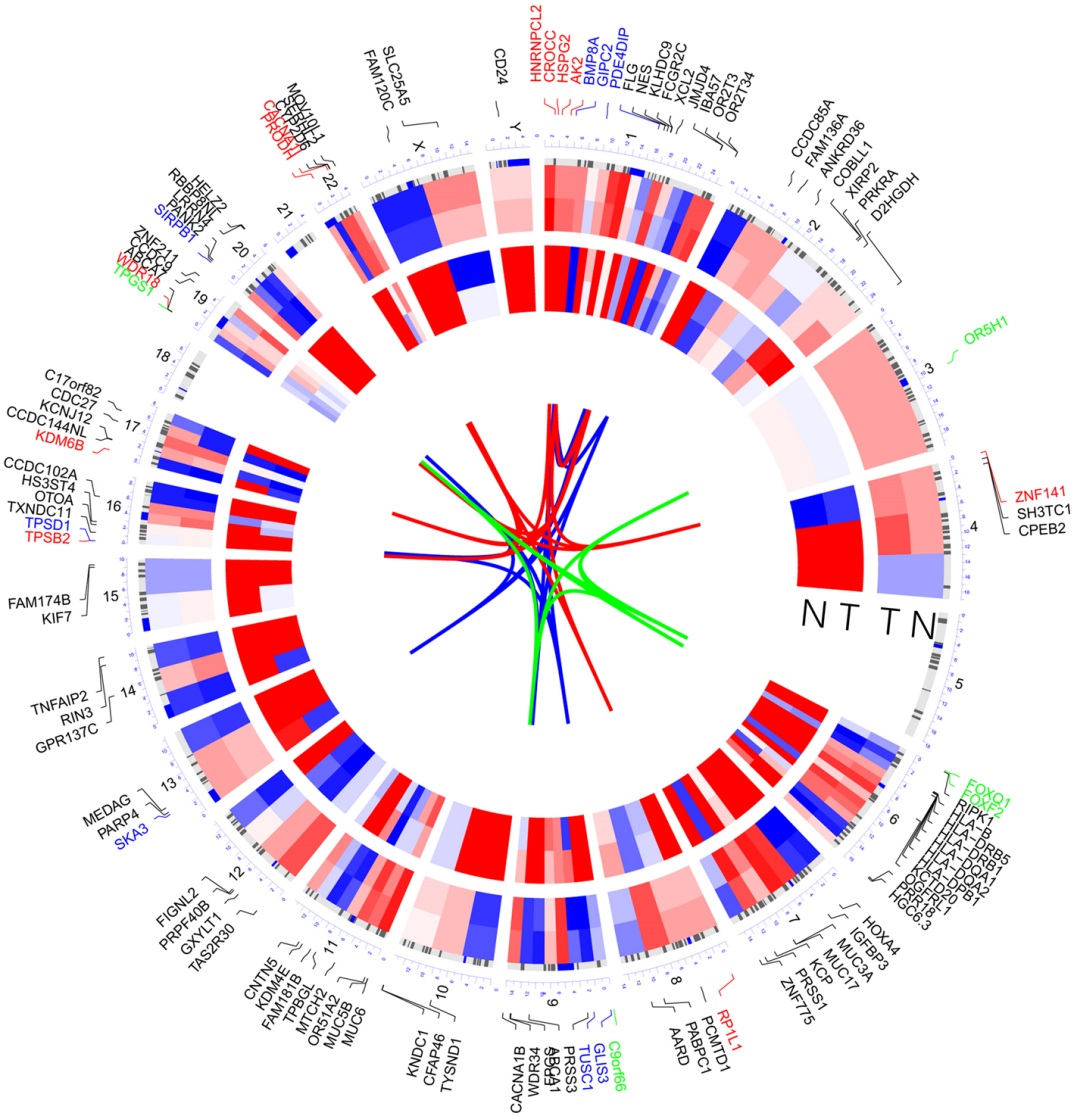
The site and the expression of unique variant genes. The outer circle shows those genes’ location on the chromosomes. The middle circle is the heat map of the gene expression. The inner circle presents the mutation frequency. In the heat map, red stands for high and blue for low. N stands for the healthy individual and T stands the diseased individual (ENKTL)

OmicCircos packages are used for the visualization of 126 unique variant genes. Figure 2 depicted the site and the expression of unique variant genes. The outer circle shows those genes’ location on the chromosomes. The middle circle is the heat map of the gene expression. The inner-circle presents the mutation frequency. In the heat map, red stands for high and blue for low.

### Functional enrichment analysis

**Fig. 3:**
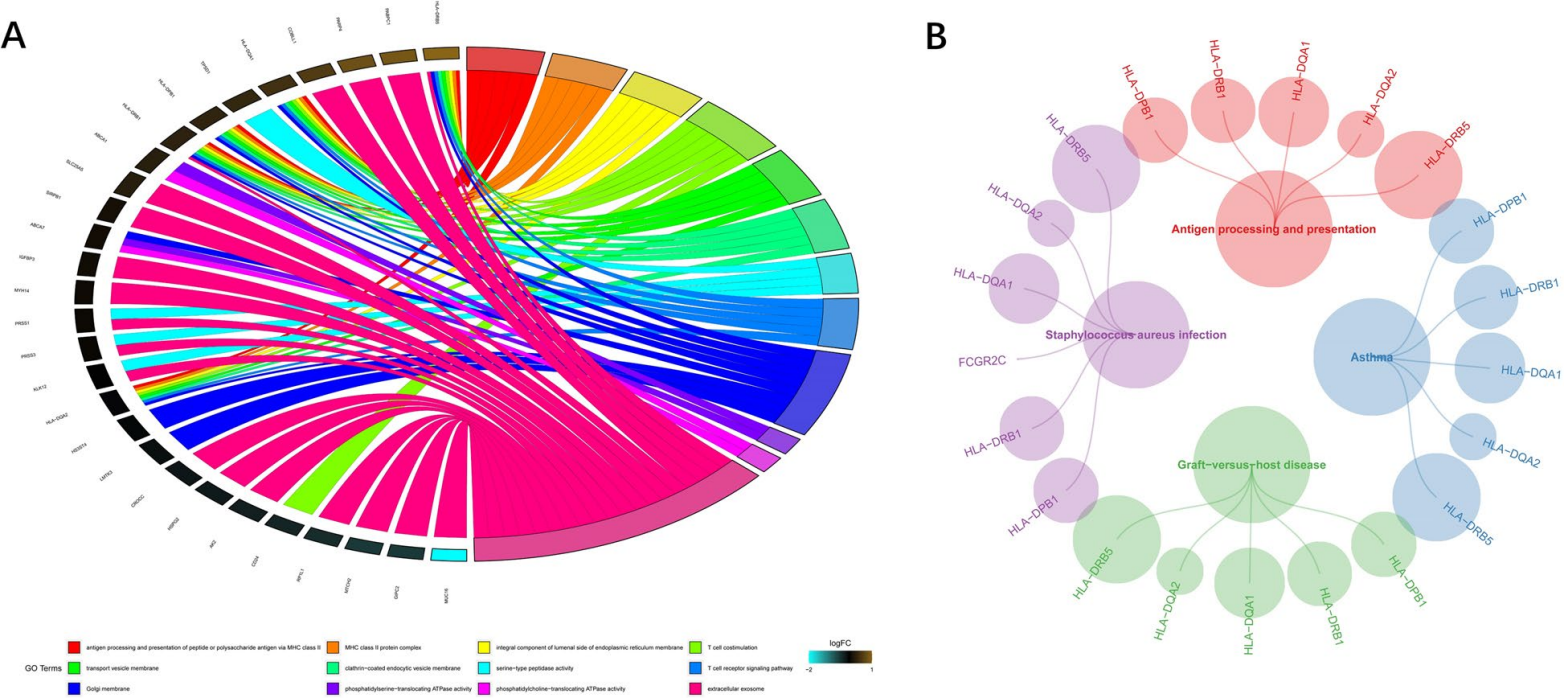
The biological function of identified unique genes were mainly enriched in immune-related biological functions and pathways. **A** Chord diagram. **B** Bubble diagram

We conducted GO and KEGG analysis to understand the biological function of identified unique genes, respectively. Chord diagram (Fig. 3A) showed that the unique genes were enriched in extracellular exosomes, Golgi membrane, T cell receptor signaling pathway, serine−type peptidase activity, clathrin−coated endocytic vesicle membrane, transport vesicle membrane, T cell costimulation, integral component of lumenal side of endoplasmic reticulum membrane, MHC class II protein complex and antigen processing and presentation of peptide or polysaccharide antigen via MHC class II. Bubble diagram (Fig. 3B) depicted that the unique genes were enriched in antigen-processing and presentation, asthma, graft-versus-host disease and staphylococcus aureus infection.

### Identification of unique genes for ENKTL

**Fig. 4:**
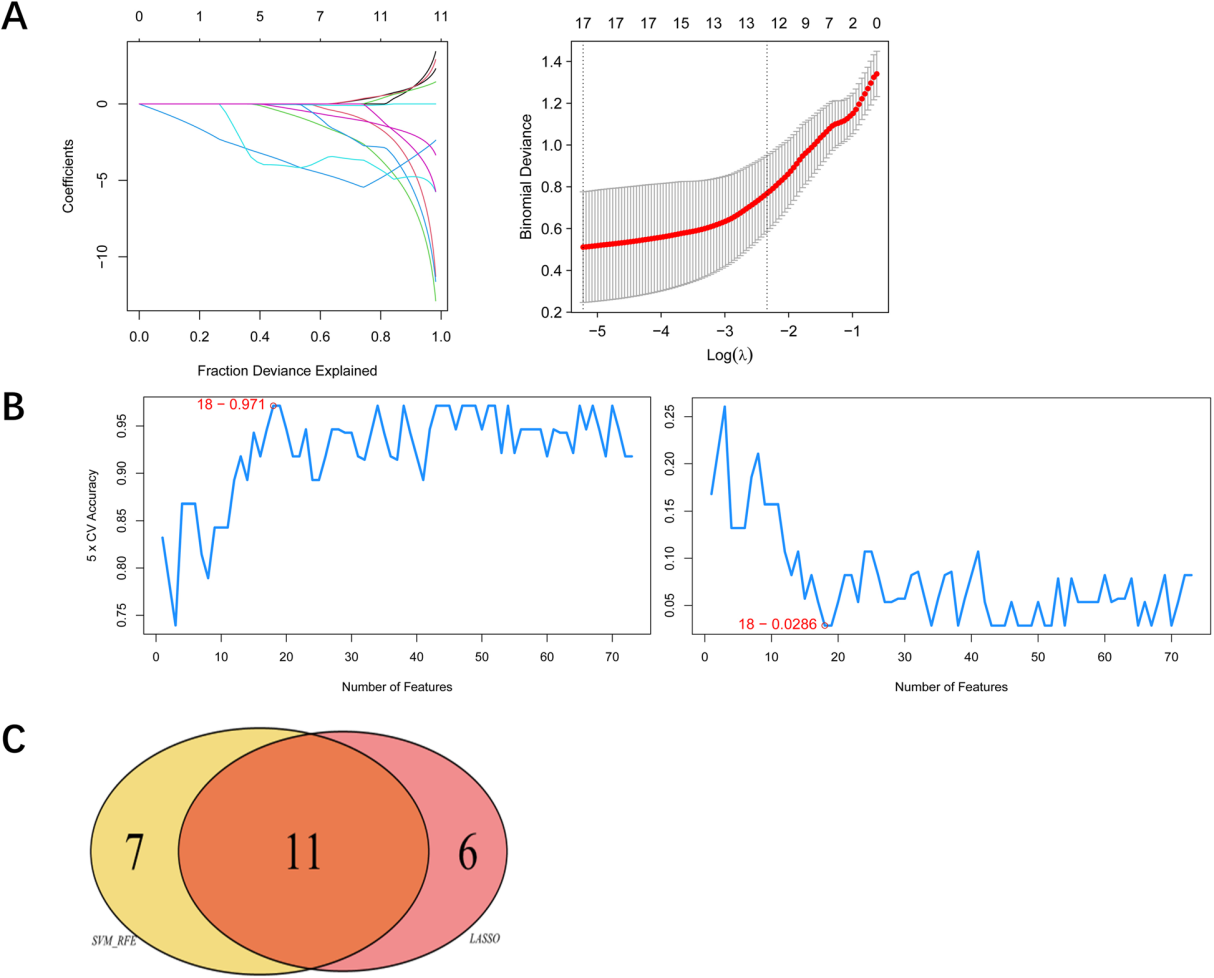
The identification of unique genes for ENKTL to distinguish tumors from normal samples. **A** The binomial classifer model is the most stable when we selected 17 genes. **B** The SVM-RFE model is intensively stable when we selected 18 genes (accuracy = 0.971) for classifying ENKTL patients and healthy individuals. **C** We identified 11 unique genes by the combination of elastic net and SVM-RFE

To find the best gene signature in the 126 unique variant genes, we constructed an elastic net. In Fig. 4A, the binomial classifier model is the most stable when we selected 17 genes. Similarly, we used SVM-RFE to identify the gene signature. In Fig. 4B, the model is intensively stable when we selected 18 genes (accuracy = 0.971) for classifying ENKTL patients and healthy individuals. By combining unique genes from the elastic net and the SVM-RFE algorithms, we identified 11 unique genes (Fig. 4C).

### GSEA and GSVA for pathway enrichment analysis

**Fig. 5:**
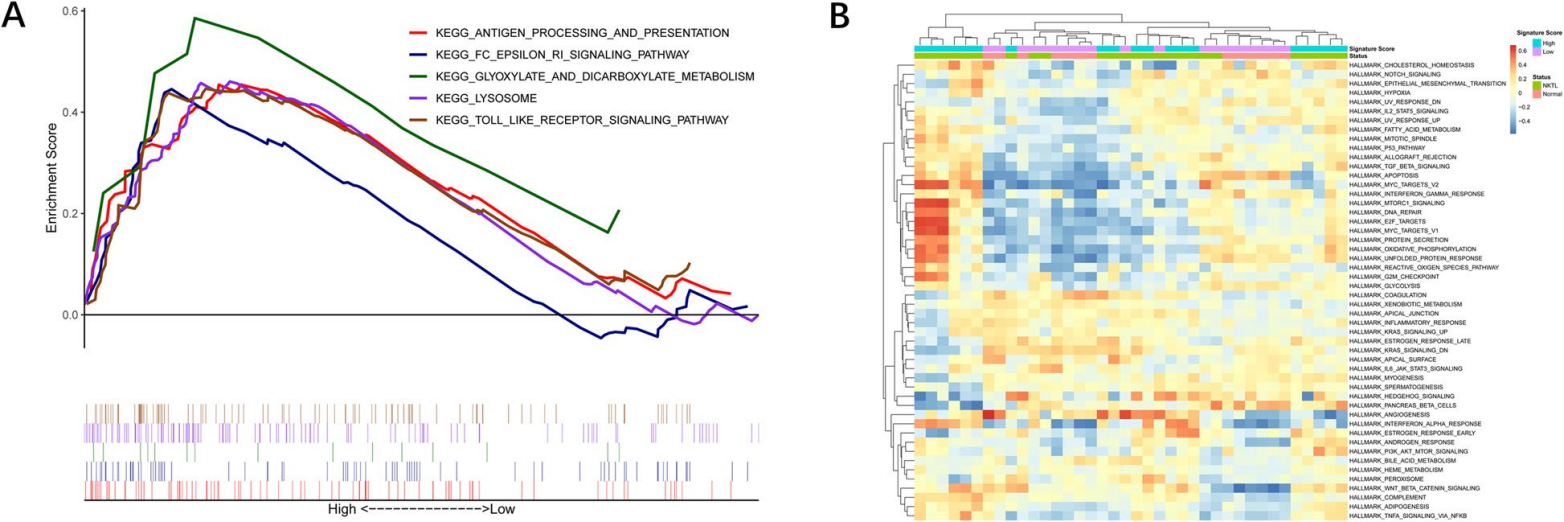
GSEA and GSVA. **A** GSEA showed that immune-related pathways were enriched in groups with high signature scores. **B** The heat map of GSVA showed that the signal pathways in the display circles were enriched in NKTL and signature groups with high scores

GSEA (Fig. 5A) indicated five immunity-related pathways with high signature scores: antigen-processing and presentation, FC-epsilon RI signaling pathway, glyoxylate and dicarboxylate metabolism, lysosome and Toll-like receptor signaling pathway. Antigen processing/presentation and Fc-related signaling pathways require the activation of antigen-presenting cells, implying that the inactivation of antigens may be a contributor for tumor cells to escape the surveillance. A synthetic toll-like receptor 4 (TLR4) agonist resulted in T-cell inflammation of the tumor microenvironment (TME) to cure lymphomas [17]. We assume that targeting the Toll-like receptor signaling pathway might be a method to treat ENKTL.

The heat map (Fig. 5B) shows GSVA results. The group with high signature scores was significantly enriched in the p53 pathway, reactive oxygen species pathway and protein secretion. The group with low signature scores was significantly enriched in coagulation, angiogenesis and myogenesis. p53 expression was associated with tumor stage and international prognostic index in patients with ENKTL [31]. p53 mutation and the upregulation of anti-apoptotic protein (survivin) favors the progression of ENKTL [32].

### Immune infiltration analysis

**Fig. 6:**
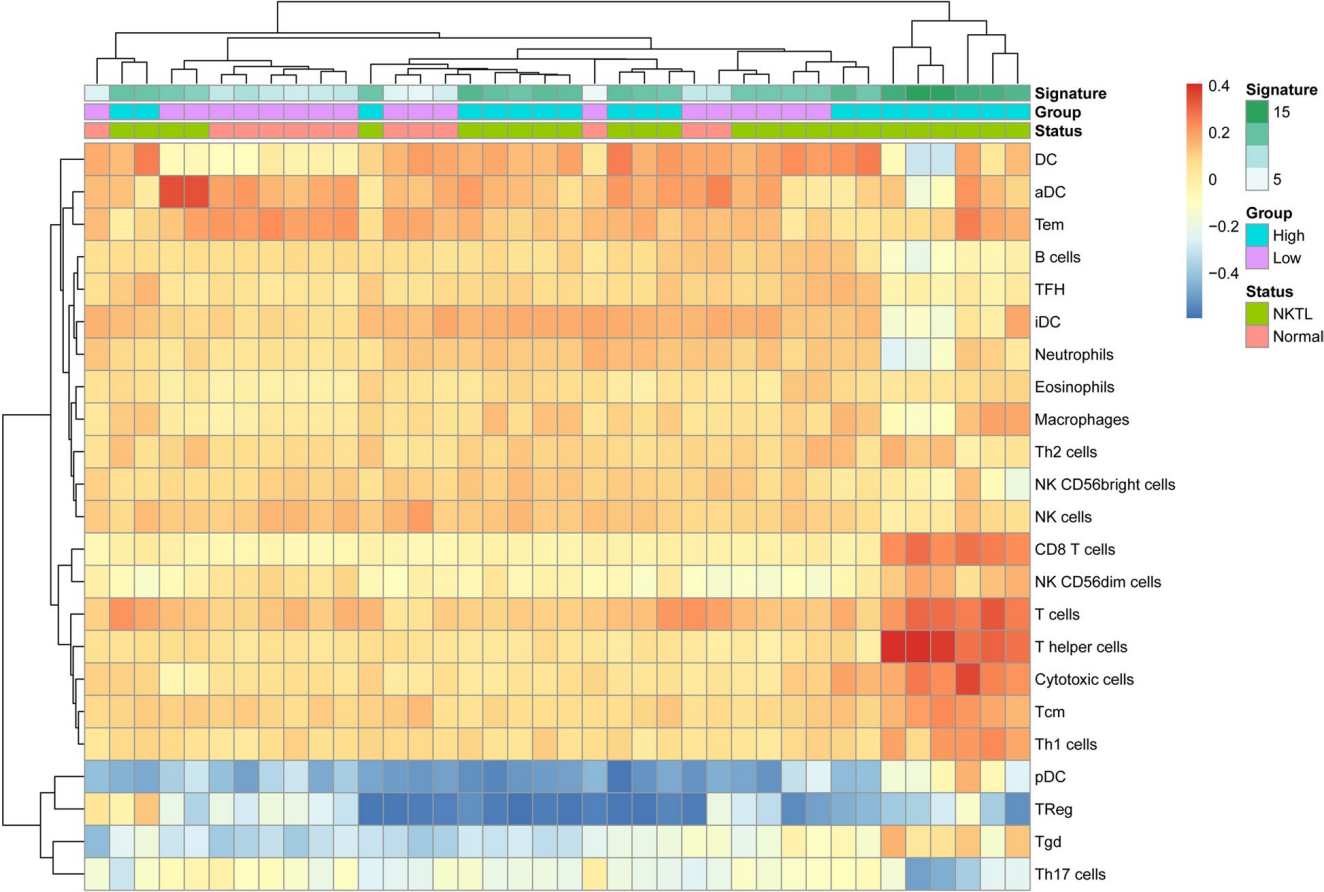
The heatmap of immune infiltration of tumor microenvironment. Red stands for high enrichment scores and Blue for low enrichment scores

The spearman correlation of unique-gene expression and corresponding immune enrichment scores were presented in Fig. 6. In ENKTL patients of the training set and the group with high signature scores, CD8^+^ T cells, NK CD56^dim^ cells, T helper cells, cytotoxic cells and central memory T cells (Tcm) are all of great infiltration. The two groups shared the same results. On the other hand, dendritic cells effector and memory T cells (Tem) are all of great infiltration in healthy individuals and the group with low signature scores.

### Validation of signature genes

**Fig. 7:**
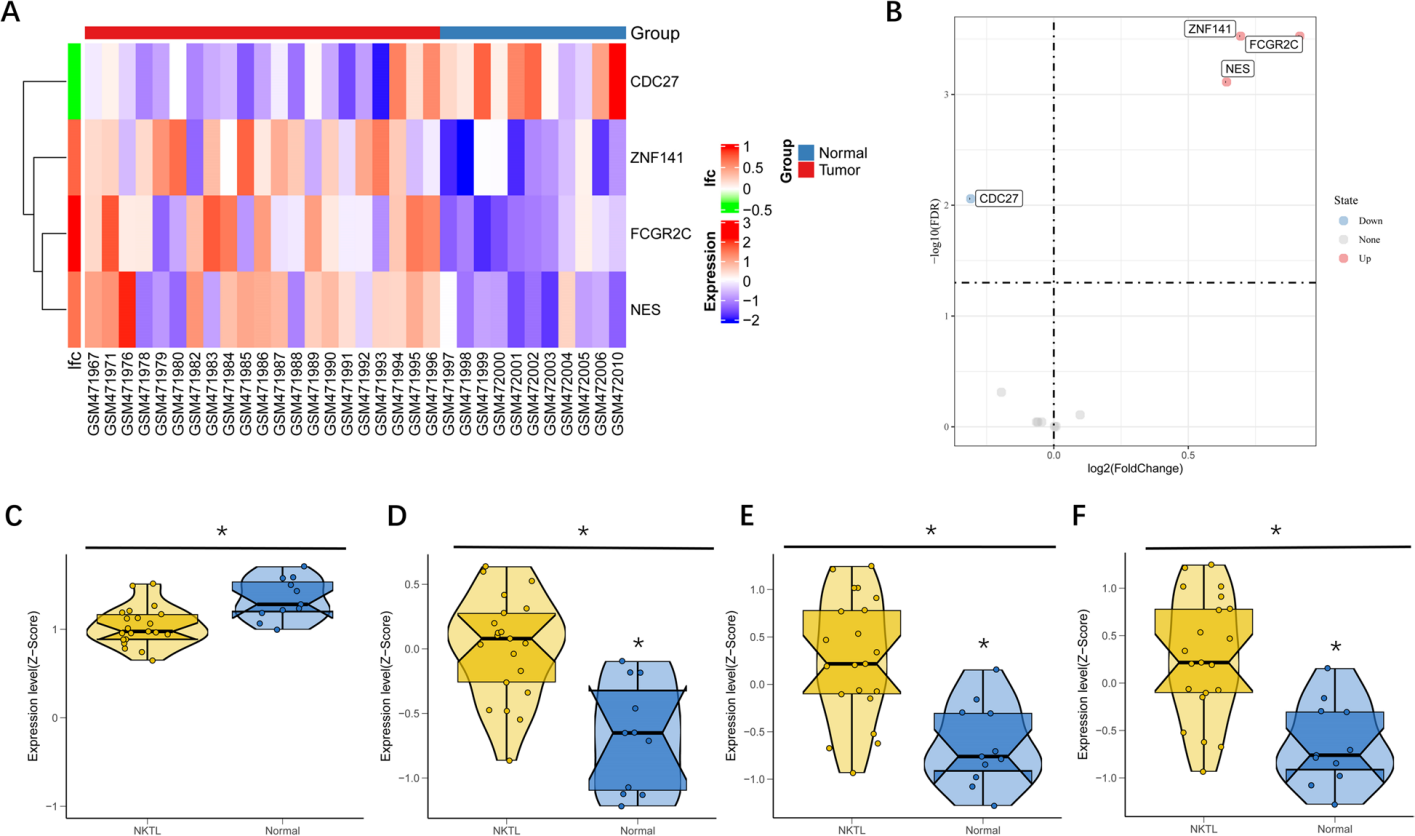
The confirmation of the accuracy of signature genes. **A** The heat map showed results of differential analysis. **B** The volcanic map showed results of differential analysis. **C** Boxviolin plot indicated the expression levels of four unique genes

We used validation sets to confirm the accuracy of our signature genes. Subsequently, the pheatmap and the volcano plot showed significantly differential genes (CDC27, ZNF141, FCGR2C and NES) in those 11 signature genes. ZNF141, FCGR2C and NES were upregulated in patients with ENKTL, while CDC27 was significantly downregulated in those patients (Fig. 7A and B). More convincingly, Boxviolin plot (Fig. 7C) indicated the expression levels of four unique genes. Consistently, the mRNA levels of ZNF141, FCGR2C and NES were higher in patients with ENKTL, while CDC27 was significantly lower.

## Discussion

ENKTL can be easily diagnosed by morphology, immunohistochemical markers and in situ hybridization. Currently, there is no standard ENKTL guideline for prevention and treatment and no retrospective study with large samples. Previous retrospective studies indicated that the therapeutic effect of advanced and recurent ENKTL is unsatisfactory. In multiple studies, corresponding prognostic factors are inconsistent [33–35]. Also, there is no prognostic molecular marker that is applied in clinical practice. Therefore, it is imperative to seek ENKTL biomarkers for treatment and prognosis. We hope that these biomarkers could accurately evaluate the prognosis of patients, promote targeted therapy in ENKTL and develop individualized treatment plans.

Several methods are used to build linear regression models. Each method is suitable for a given dataset with different features. The response variable (*n*) and the predictive variable (*p*) reflect the bias of these linear regression models. Our study consists of 38 samples. Elastic networks and SVM were used to screen specific target genes from unique variants to distinguish tumors from normal samples [36]. Elastic networks are suitable for our data that independent variables are much less than dependent variables (*n* < < *p*). We screened out 11 gene expression signatures for prediction. These are CDC27, MOV10L1, CROCC, RP1L1, ZNF141, FCGR2C, NES, CCDC9, TPSD1, CACNA1I, BMP8A.

With algorithms, scientists have applied machine learning to predict diagnosis, prognosis and therapeutic efficacy in lymphoma [37–39]. For example, Hyungsoon et al. developed an automated device for the molecular diagnosis of aggressive lymphomas. They validated nodal lesions suspicious for lymphoma in 40 patients. The device can be portable to classify benign and malignant tumors [37]. Moreover, Shipp et al. applied supervised learning to identify cured diseases and fatal/refractory diseases. Specifically, the algorithm classified patients with different five-year survival rates and prognostic indexes (IPI) into two groups for outcome prediction, respectively [38]. Besides, Julkunen et al. constructed a machine learning framework (comboFM) to predict the responses of drug combinations. They found synergistic action in the combination of an anaplastic lymphoma kinase inhibitor (crizotinib) and a proteasome inhibitor (bortezomib) in lymphoma [39]. The performance stability of these models could be further compensated by choosing the study population, classifying pathological type and enlarging sample size.

Importantly, our data is from a pair of identical twins. One is diagnosed with ENKTL, while the other is healthy. We collected a cancerous sample from the ENKTL patient and a non-cancerous sample from the healthy one. We screened out unique mutant genes from the cancerous patient by setting the healthy one as control, which suggests that some of these mutant genes might be potential pathogenic genes. Our result is more convincing to explain the alterations in ENKTL pathogenesis. Next, our study performed an elastic analysis of ENKTL patients from international multi-platforms with SVMs for improved accuracy. Compared with linear mixed effect models (NONMEMs) and neural network models, SVMs solve problems better, including model selection, over-learning, nonlinear and dimension disaster and local minimum. According to the limited sample information, SVMs can find the best compromise between the complexity and learning ability of the model to obtain the best generalization. The method enables our predictive models appliable in predicting ENKTL.

Mechanically, the tumorigenesis and invasion of ENKTL are complicated. We comprehensively analyzed the molecular network by using GO and KEGG enrichment analysis. The purpose is to elucidate the pathogenesis of ENKTL and find sites for targeted therapy. Through the functional enrichment of unique variant genes, we understand the biological processes of these genes in ENKTL. Figure 3A indicated that extracellular exosomes were significantly correlated with ENKTL. A study showed similar results that the upregulated exosomal miRNA was a biomarker to identify ENKTL patients with treatment failure [35]. Exosomal miRNAs might be a biomarker to indicate therapeutic efficacy. Besides, we found that Golgi membrane, clathrin−coated endocytic vesicle membrane, transport vesicle membrane, endoplasmic reticulum membrane were all participated in the development of ENKTL, according to Fig. 3A. Latent membrane protein 1 (LMP1) is a stimulant of NKTL progression, which upregulates eukaryotic translation initiation factor 4E (eIF4E) mediated by the NF-κB pathway [40]. We hypothesized that these membrane-related mechanisms are involved in the activation of the tumorgenesis pathway, serving as an indicator of tumor progression. Other immunological signals (T cell receptor signaling pathway and phosphatidylcholine /phosphatidylserine-translocating ATPase activity) and complexes (MHC class II) are involved in ENKTL. A study identified the expression of T-cell receptors in ENKTL and the re-arrangement of T-cell-receptor genes [41]. The inhibition of ATPase activity and the regulation of MHC class II might be potential sites for targeted therapy.

Additionally, several eregulated cellular signaling networks have been extensively investigated in ENKTL. Janus kinase/signal transducer and activator of transcription (JAK/STAT) pathway is the first representative. Compared with normal NK cells, proteins in the JAK/STAT pathway are differentially expressed in ENKTL cells [13, 42]. Platelet-derived growth factor receptor-α (PDGFR-α) pathway is another activated pathway in ENKTL and is correlated with cellular biological functions. Huang et al. used a tyrosine kinase inhibitor (imatinib mesylate) to inhibit the growth of the PDGFRα-overexpressing ENKTL cell line (MEC04) [13]. NOTCH-1 signaling pathway involves Notch 1 and Notch 2 which synergistically regulate the differentiation and function of NKT cells [43]. Similarly, Huang et al. used two NOTCH-1 inhibitors to hinder NK cell growth [13]. Figure 5A indicated that these potential pathways are related to antigen processing and the Fc epsilon RI-mediated signaling pathway. Stimulatory antigens might be processed for presentation. Precessed antigens could bind to the extracellular domain of the α chain of Fc epsilon RI to initiate intracellular signals. Furthermore, our results show the involvement of metabolic pathways, lysosomal pathways and Toll-like receptor pathways. JAK/STAT pathway, PDGFR-α pathway and NOTCH-1 participate in the energy metabolism and lysosomal activities. Our findings are consistent with the previous study.

We depicted the landscape of ENKTL and identified a series of targetable genes. Among them, CDC27 (Cell division cycle 27), ZNF 141 (Zinc finger protein141), Fc gamma receptor 2C (FCGR2C) and NES (nestin) are four promising candidates. Both the upregulation of ZNF141, FCGR2C and NES and the downregulation of CDC27 were associated with robust dendritic cell (DC) and T cell infiltration. Our deduction may be that ENKTL-associated proteins can be processed by DCs and presented to CD8^+^ T cells in the event of adequate other kinds of T cell infiltration to induce an immune attack. On the one hand, we analyzed these candidates functionally by GO enrichment analysis, KEGG enrichment analysis, GSEA and GSVA. On the other hand, their potential function in tumors was also investigated in previous literature. First, CDC27 is a significant subunit responsible for promoting anaphase. High levels of CDC27 were witnessed in T-lymphoblastic lymphoma (T-LBL). It facilitated proliferation, G1/S transition, protein upregulation (cyclin D1, CDK4 and PD-L1) and the inhibition of apoptosis [44]. Next, ZNF 141 encodes gene mapping and is related to chromosomal aneusomy syndromes. Its defect causes developmental disorders, involving some transcriptional regulators. Chromosomal aneusomy is one of the common genetic features of malignant tumor cells. Fetal death is a common outcome of chromosomal aneusomy [45]. Then, FCGR2C correlates with Fc gamma receptors of low-affinity immunoglobulins. It is a transmembrane glycoprotein located on the surface of immune cells and participates in phagocytosis and clearance of immune complexes [46]. NES is a kind of intermediate filament protein which is used as a marker of neural stem cells and progenitor cells in the central nervous system and a marker of endothelial cells. As for cancer, nestin exists in cancer stem-like cells and poorly differentiated cancer cells [47].

While our study was the first large-scale data analysis focusing gene signatures in patients with ENKTL, several limitations were noticed. We obtained a number of NKTL’s unique variant genes from the sequencing data of a pair of twins. Due to the limited number of samples, we selected the training set and validation sets of ENKTL from the public library to explore the predictive efficacy of these unique variant genes for ENKTL. We hope to find out a set of the most important signature genes for ENKTL. First, we conducted WGS, instead of detecting the mRNA level of these genes. Hence, the transcriptional level of gene expression is lack of validation in twins. Second, in multiple platforms, analyzing large cohort results in batch effects which are caused by different time, operators, reagents and instruments. Finally, a limited number of patients is another limitation. Our patients are a pair of twins. The best identification results need more data for validation and confirmation.

## Conclusion

We conducted WGS for sequencing to identify unique variant genes from the peripheral blood samples of an ENKTL patient and a healthy individual. By analyzing the database, we demonstrated CDC27, MOV10L1, CROCC, RP1L1, ZNF141, FCGR2C, NES, CCDC9, TPSD1, CACNA1I, BMP8A as unique genes of ENKTL. Their involvement of biological activity and immune filtration was associated with ENKTL tumorigenesis and progression. ENKTL was caused by antigen processing/presentation pathway, Fc epsilon RI signaling pathway, glyoxylate and dicarboxylate metabolism pathway, lysosome pathway and Toll-like receptor signaling pathway. Finally, our study concluded that ZNF141, FCGR2C, NES and CDC27 are promising ENKTL gene signatures. These four genes showed good predictive efficacy in the validation set, suggesting that they are convincing signature genes for ENKTL.

## Data Availability

From the peripheral blood of a pair of twins, we applied a whole-genome shotgun (WGS, Beijing Boao Biological Co., Ltd) for sequencing to identify unique variant genes. We downloaded a training dataset (GSE 80632) from Gene Expression Omnibus (GEO) (https://www.ncbi.nlm.nih.gov/gds/) based on GPL13158 Affymetrix HT HG-U133+ PM Array Plate.

## Abbreviations

ENKTL: Extranodal NK/T-cell lymphoma
RMA: Robust Multi-array Analysis
EBV: Epstein-Barrvirus
PD-1: Programmed cell death protein 1
PD-L1: Programmed cell death protein ligand 1
EGFR: Epidermal growth factor receptor
ALK: Anaplastic lymphoma kinase
GEP: Gene expression profiling
γδ-PTCLs: Gamma delta-peripheral T cell lymphomas
MCR: Minimal common region
SVMs: Support-vector machines
WGS: Whole-genome shotgun
GEO: Gene Expression Omnibus
GO: Gene Ontology
KEGG: Kyoto Encyclopedia of Genes and Genomes
SVM-RFE: Support Vector Machine-Recursive Feature Elimination
GSEA: Gene set enrichment analysis
GSVA: Gene Set Variation Analysis
ssGSEA: Single-sample gene set enrichment analysis
TLR4: Toll-like receptor 4
TME: Tumor microenvironment
LMP1: Latent membrane protein 1
eIF4E: Eukaryotic translation initiation factor 4E
PDGFR-α: Platelet-derived growth factor receptor-α
CDC27: Cell division cycle 27)
ZNF 141: Zinc finger protein141
FCGR2C: Fc gamma receptor 2C
NES: Nestin
DC: Dendritic cell
T-LBL: T-lymphoblastic lymphoma
DNBs: DNA Nanoballs
